# Osteomalacia and Vitamin D Status: A Clinical Update 2020

**DOI:** 10.1002/jbm4.10447

**Published:** 2020-12-21

**Authors:** Salvatore Minisola, Luciano Colangelo, Jessica Pepe, Daniele Diacinti, Cristiana Cipriani, Sudhaker D Rao

**Affiliations:** ^1^ Department of Clinical Internal, Anesthesiological and Cardiovascular Sciences, Sapienza University of Rome Rome Italy; ^2^ Bone and Mineral Research Laboratory, Division of Endocrinology Diabetes & Bore and Mineral Disorders, Henry Ford Hospital Detroit MI USA

**Keywords:** HISTOMORPHOMETRY, INTESTINAL DISEASES, MUSCLE WEAKNESS, OSTEOMALACIA, VITAMIN D

## Abstract

Historically, rickets and osteomalacia have been synonymous with vitamin D deficiency dating back to the 17th century. The term osteomalacia, which literally means soft bone, was traditionally applied to characteristic radiologically or histologically documented skeletal disease and not just to clinical or biochemical abnormalities. Osteomalacia results from impaired mineralization of bone that can manifest in several types, which differ from one another by the relationships of osteoid (ie, unmineralized bone matrix) thickness both with osteoid surface and mineral apposition rate. Osteomalacia related to vitamin D deficiency evolves in three stages. The initial stage is characterized by normal serum levels of calcium and phosphate and elevated alkaline phosphatase, PTH, and 1,25‐dihydroxyvitamin D [1,25(OH)_2_D]—the latter a consequence of increased PTH. In the second stage, serum calcium and often phosphate levels usually decline, and both serum PTH and alkaline phosphatase values increase further. However, serum 1,25(OH)_2_D returns to normal or low values depending on the concentration of its substrate, 25‐hydroxyvitamin D (25OHD; the best available index of vitamin D nutrition) and the degree of PTH elevation. In the final stage, hypocalcemia and hypophosphatemia are invariably low with further exacerbation of secondary hyperparathyroidism. The exact,or even an approximate, prevalence of osteomalacia caused by vitamin D deficiency is difficult to estimate, most likely it is underrecognized or misdiagnosed as osteoporosis. Signs and symptoms include diffuse bone, muscle weakness, and characteristic fracture pattern, often referred to as pseudofractures, involving ribs, scapulae, pubic rami, proximal femurs, and codfish‐type vertebrae. The goal of therapy of vitamin D‐deficiency osteomalacia is to alleviate symptoms, promote fracture healing, restore bone strength, and improve quality of life while correcting biochemical abnormalities. There is a need for better understanding of the epidemiology of osteomalacia. Simplified tools validated by concurrent bone histology should be developed to help clinicians promptly diagnose osteomalacia. © 2020 The Authors. *JBMR Plus* published by Wiley Periodicals LLC. on behalf of American Society for Bone and Mineral Research.

## Introduction

Historically, rickets and osteomalacia have been synonymous with vitamin D deficiency dating back to the 17th century. The term osteomalacia, which literally means soft bone, was traditionally applied to characteristic radiologically or histologically documented skeletal disease and not just based on clinical or biochemical features. Osteomalacia, viewed principally as a mineralization defect, can manifest in several types, which differ from one another by the characteristic relationships of osteoid (ie, unmineralized bone matrix) thickness both with osteoid surface and adjusted mineral apposition rate. Strictly speaking, an increase only in osteoid surface or osteoid thickness is really not osteomalacia as traditionally defined. Excess osteoid accumulation (surface and/or volume) can occur in several conditions: states of high bone turnover (ie, hyperparathyroidism, hyperthyroidism) enzyme defects (ie, hypophosphatasia), matrix disorders (ie, fibrous dysplasia, fibrogenesis imperfecta ossium). Currently, most experts agree that in the absence of characteristic radiological findings of pseudofractures, osteomalacia can only be diagnosed by transiliac bone biopsy (after tetracycline double‐labeling), when osteoid volume is >5%, uncorrected osteoid thickness is ≥15 μm, and the mineralization lag time is >100 days.^(^ ^1^, ^2^ ^)^ Furthermore, in its early stages, osteomalacia requires histomorphometric evaluation before any irreversible cortical bone loss and skeletal deformities have occurred^(^ ^3^ ^)^ by the time osteomalacia is clinically suspected.^(^ ^1^ ^)^

Therefore, it is clear that any further discussion concerning osteomalacia and vitamin D status in 2020 cannot disregard the radiological and histological basis of the disease. However, this is difficult for several reasons: bone biopsy is an invasive and painful procedure not well‐accepted by patients; very few clinicians and investigators in the world are able to perform transiliac bone biopsy on an ambulatory basis; and the histological evaluation requires a specialized laboratory that can process undecalcified bone sections and perform detailed histomorphometry. Recently, noninvasive diagnostic criteria have been proposed to circumvent the challenges related to bone histological criteria based on a constellation of clinical (diffuse bone pain and muscle weakness), radiological (reduced BMD, Looser's zone or pseudofractures on X‐ray, or diffuse multiple uptakes by bone scintigraphy), and biochemical findings.^(^ ^4^ ^)^ However, these criteria have not been rigorously validated and may not be widely applicable.

## Prevalence

An exact or even an approximate prevalence of osteomalacia caused by vitamin D deficiency in the world is very hard to define because the condition is often asymptomatic in most cases, especially in the elderly, or remains underrecognized in many cases. However, on a global scale, vitamin D deficiency is by far the most common cause of osteomalacia. Although rickets occurs only in growing children and adolescents before epiphyseal fusion, osteomalacia, as traditionally defined, occurs both in children and adults. Thus, it can be assumed that osteomalacia is quite prevalent in parts of the world where nutritional rickets is common, although histological confirmation of osteomalacia in children and adolescents with rickets is lacking. Furthermore, recent mass migration into industrialized countries poses a different problem. Because plentiful sunshine allows normal cutaneous vitamin D production in the country of origin, individuals may become at risk for vitamin D deficiency when they migrate to northern Europe or North America, where the ultraviolet spectrum of sunlight is lacking for most or part of the year.

The underappreciation of real prevalence of osteomalacia in the world is highlighted in two old bone biopsy studies. In the first study, Hordon and Peacock examined 78 unselected patients (68 women and 10 men) at the time of acute proximal femoral fracture and found that the prevalence of osteomalacia increased with age: In those over the age of 90 years, it occurred in 29% of patients.^(^ ^5^ ^)^ In the other Italian study, histomorphometry of bone biopsies of 45 patients with a clinical diagnosis of osteoporosis, histological osteomalacia, as defined above, was found in 4.4%, whereas another 8.9% was termed as “osteoporomalacia.”^(^ ^6^ ^)^ Other reports with less‐stringent criteria for osteomalacia have reported similar prevalence. Nevertheless, it is clear that both the incidence in an at‐risk population and its prevalence on a global scale are probably vastly underestimated.

## Biochemical and Histological Evolution of Osteomalacia

From a biochemical point of view, osteomalacia caused by vitamin D deficiency evolves in three stages: an initial stage characterized by normal serum levels of calcium and phosphate and elevated levels of alkaline phosphatase, serum PTH, and 1,25‐dihydroxyvitamin D [1,25(OH)_2_D]—the latter as a consequence of increased PTH. At this stage, bone histomorphometry reveals only the effects of excess PTH without a mineralization defect. In the second stage, serum calcium and often phosphate levels usually decline, and both serum PTH and alkaline phosphatase values increase further; however, serum 1,25(OH)_2_D returns to normal or low values depending on the concentration of its substrate and the degree of PTH elevation. There is some evidence of impaired mineralization as assessed by tetracycline‐labeled bone histomorphometry. In the final stage, hypocalcemia and hypophosphatemia are invariably low together with further exacerbation of secondary hyperparathyroidism and mineralization of bone matrix ceases to occur.

## Etiology and Pathophysiology

There are four pathogenic mechanisms for the development of osteomalacia: (i) vitamin D deficiency or resistance, (ii) calcium deficiency rickets (presumably osteomalacia) independent of vitamin D nutritional status,^(^ ^7^ ^)^ (iii) phosphate depletion caused by a primary or secondary increase in fibroblast growth factor‐23 secretion or other phosphatonins,^(8^ ^)^ and (iv) inhibition of mineralization caused by the toxic effects of various drugs. In the following section, we exclusively address the osteomalacia that is directly related to vitamin D deficiency. (Other aspects of vitamin D are discussed in related articles in this special issue.)

**Fig 1:**
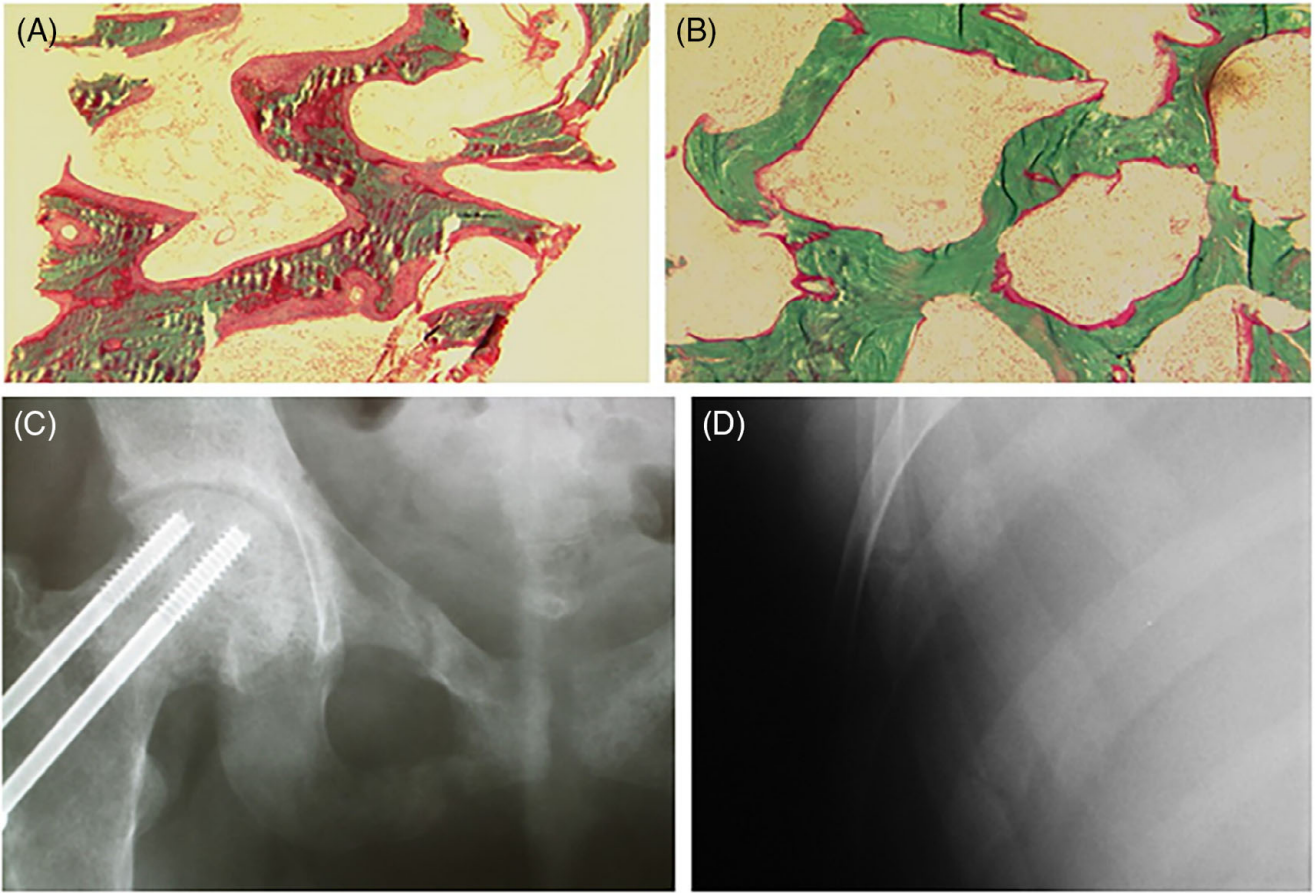
Bone histomorphometry in a patient with osteomalacia. There are wide and extensive osteoid before treatment (*A*) and almost complete disappearance of osteoid after treatment with vitamin D (*B*); from reference (1). A patient with osteomalacia caused by celiac disease. Pelvis with lucency traversing the superior and inferior pubic rami, with varying degrees of callus formation (*C*); transverse lucencies of two ribs (*D*). The patient was finally diagnosed as having celiac disease.

Based on the underlying mechanism responsible, the causes of osteomalacia from vitamin D deficiency can be broadly categorized into two large groups: extrinsic (environmental, secular, or behavioral), and intrinsic (specific to the individual at risk; Table 1). The extrinsic causes are inadequate dietary intake of vitamin D, decreased sunlight exposure, avoidance of sunlight, use of sunscreens with a high sun‐protection factor, fully covering clothing for cultural or religious reasons, and dark skin pigmentation. Apart from the decreased cutaneous production of vitamin D with aging, vitamin D malabsorption is the most common intrinsic cause of osteomalacia. Diseases resulting in osteomalacia from vitamin D malabsorption include gluten enteropathy (the most common), gastrectomy, small intestinal disease, resection or bypass, primary biliary cholangitis, and pancreatic insufficiency. The relative frequency of osteomalacia in various gastrointestinal (GI) disorders is difficult to ascertain. Furthermore, the issue is complicated by the fact that because of concomitant malabsorption of calcium and other nutrients, the clinical and histological features may span from osteopenia through osteoporosis to osteomalacia (Fig. 1).

**Table 1:** Causes of vitamin D Deficiency Osteomalacia*

**Extrinsic (extraneous to the individual such as environmental, secular or behavioral)** Inadequate dietary intake of vitamin D Decreased exposure or avoidance of sunlight Use of sunscreens (especially >8 SPF) Fully covered garbs (veil, hijab, burqa, Indian Saree etc.) Dark skin pigmentation
**Intrinsic (within the individual)** Advancing age with decreased cutaneous production of vitamin D Morbid Obesity Malabsorption due to various gastrointestinal disorders Gastrectomy (partial, total, or gastric‐bypass procedures) Small intestinal disease, resection or bypass Gluten enteropathy (Celiac sprue) Primary Biliary Cholangitis (uncommon) Pancreatic insufficiency including cystic fibrosis (uncommon) Impaired or genetically defective vitamin D‐25‐hydroxylase Immaturity Neonatal hepatitis Cirrhosis of the liver Impaired or genetically defective 25‐hydroxyvitamin D‐1α hydroxylase Genetic defect (Vitamin D dependent rickets type‐1A) Chronic renal failure
**“Acquired” vitamin D deficiency (Increased catabolism or metabolic clearance)** Anticonvulsants Calcium malabsorption with secondary hyperparathyroidism Primary hyperparathyroidism Paget's disease of bone (presumed excess consumption in Pagetic bones)

* Modified from reference 10 (Bhan et al)

From a pathophysiological point of view, prolonged vitamin D deficiency (perhaps exacerbated by calcium deficiency) decreases serum calcium with compensatory increases in serum PTH levels. The increased PTH levels, which act on bone and kidney, have the ultimate effect of resetting the system to raise serum calcium towards normal. As such, elevated serum PTH levels can be considered a pathognomonic hallmark in the vast majority—but not necessarily all cases—of patients with vitamin D deficiency. Therefore, the biochemical constellation includes a tendency for low serum calcium levels (adjusted for albumin), low serum phosphate levels (caused by phosphaturic effect of high PTH levels in the proximal renal tubule), and elevated serum alkaline phosphatase (owing to the effect on bone). This last biochemical finding reflects an increase in compensatory osteoblastic activity. Sustained PTH hypersecretion, together with a lack of calcium supply, results in reduced BMD (often misinterpreted as having osteoporosis) and structural competence of the skeleton resulting in fractures, the most characteristic being pseudofracture (Looser's zones). This is manifested by a radiolucent line through the cortical plate, perpendicular to the long axis of bone, often with sclerosis seen at the margins. From a histological point of view, the evolution of osteomalacia begins with a condition of secondary hyperparathyroidism (stage 1) that progresses to some mineralization defect (stage 2), and finally with the finding of frank osteomalacia (stage 3).^(^ ^1^ ^)^

## Clinical Manifestations of Osteomalacia

Clinical manifestations of osteomalacia primarily include diffuse bone pain and tenderness,^(^ ^9^ ^)^ muscle weakness, and fragility fractures. Skeletal symptoms are quite nonspecific and common to bone and non‐bone–related diseases. From a mechanical point of view, incompletely mineralized bone is weaker but more flexible, resulting in bowing of the long bones of the lower extremities. It has been shown that incompletely mineralized fibrils of rachitic mice are more extensible and less stiff, thus exhibiting greater strain and bendability.^(^ ^10^ ^)^ Finally, because of reduced mineral content and skeletal strength, fractures may occur in both axial and appendicular skeleton. The finding of a pseudofracture (Looser's zone) is almost always diagnostic of osteomalacia in the right clinical setting.

Myopathy is also a common manifestation of osteomalacia with a prevalence of 44% to 100% depending upon the degree and duration of vitamin D deficiency. Clinically, patients complain of proximal muscle weakness, mostly affecting the muscles of the thigh and knee joints, and have a characteristic waddling gait because of the inability to lift the extremity off the ground.^(^ ^11^, ^12^ ^)^ In some cases, the muscle weakness is so severe that mobility is very limited. In extreme cases, the patient is bedridden—mimicking paralysis. As previously emphasized, muscular complaints can be vague and slowly progressive; it may take many years to make a final diagnosis of vitamin D deficiency and osteomalacia. Patients may seek the advice of clinicians from other disciplines, such as rheumatologists, orthopedists, and neurologists, before being seen by a bone and mineral specialist.^(^ ^13^ ^)^ Despite being a prominent symptom of vitamin D deficiency, the pathogenesis of muscular manifestation remains poorly understood. Also, the relative contribution of hypocalcemia, hypophosphatemia, and elevated PTH levels to muscular manifestations of rickets and osteomalacia is not entirely clear, although vitamin D deficiency alone without elevated PTH levels has been reported.

## Diseases Associated With Osteomalacia

There are a number of diseases that have been potentially linked to vitamin D deficiency osteomalacia; however, their true prevalence is uncertain for several reasons including: (i) a lack of histological documentation, (ii) a misdiagnosis as the more prevalent metabolic bone disease—osteoporosis, and (iii) a lack of awareness by practicing physicians and specialists. In the next section, we will address the most important of these clinical conditions, with an emphasis on the relevant aspects pertaining to the topic of this article.

### Bariatric surgery

Bariatric surgery has been successfully employed for long‐term weight control and to improve multiple medical conditions.^(^ ^14^ ^)^ At present, sleeve gastrectomy and Roux‐en‐y gastric bypass are the most commonly used techniques. Both procedures, but especially the latter, result in vitamin D malabsorption, which can cause osteomalacia if prolonged. Accordingly, the American Society for Metabolic and Bariatric Surgery guidelines recommend vitamin D supplementation with variable doses.^(^ ^15^ ^)^

Unfortunately, there are only a few studies documenting the presence of histological osteomalacia in these patients.^(^ ^16^, ^17^ ^)^ A study carried out in obese adults undergoing biliopancreatic diversion with duodenal switch before and four years after surgery found increased osteoid volume and decreased cortical thickness.^(^ ^18^ ^)^ An important point to consider is that after bariatric surgery, vitamin D is malabsorbed. Indeed, a number of studies found that despite a variety of vitamin D supplementation regimens, mean serum 25OHD levels remain below 30 ng/mL (the threshold suggested by the Endocrine Society), even though the majority of the study population reached the 25OHD level of 20 ng/mL as recommended by the Institute of Medicine.^(^ ^19^ ^)^

### Antiepileptic medications

A number of studies in the past reported the finding of osteomalacia in adults with epilepsy. It should be noted that most of these investigations were carried out in institutionalized settings: an issue that could have constituted a confounding factor. Therefore, the relative contribution of vitamin D deficiency to the pathogenetic aspects described below is uncertain. Osteomalacia was ascribed to low vitamin D secondary to cytochrome p450 liver enzyme‐inducer antiepileptic medications; however, other factors such as poor nutrition and low sun exposure might have contributed. Initially, the mechanism for osteomalacia in patients taking antiepileptic drugs was ascribed to the metabolism of vitamin D and to inactive forms by drugs such as phenytoin, which can induce the cytochrome p450 system. Recently, the nuclear pregnane‐X receptor has been implicated in the development of osteomalacia in association with phenobarbital use. Indeed, one study reported that phenobarbital upregulates 25‐hydroxyvitamin D(3)‐24 hydroxylase gene expression in vitro by this mechanism.^(^ ^20^ ^)^ However, histomorphometric studies are missing in this condition. One study of institutionalized patients, who sustained fractures in the preceding year, showed increased resorptive activity of trabecular bone compared with controls, and an increase of osteoid suggestive of osteomalacia.^(^ ^21^ ^)^ Today, the real prevalence of osteomalacia in patients taking antiepileptic medications and the relative contribution of vitamin D deficiency remain to be determined; furthermore, no data exist about the possible direct and indirect impact of new antiepileptic drugs (oxcarbazepine, gabapentin, levetiracetam, lamotrigine) on vitamin D (ie, cholecalciferol or ergocalciferol) metabolism.

### Chronic kidney disease

In 2005,^(^ ^22^ ^)^ the Kidney Disease Improving Global Outcomes (KDIGO) Working Group simplified the complex nomenclature of renal osteodystrophy and aligned with the standard nomenclature recommended by the American Society for Bone and Mineral Research.^(^ ^23^ ^)^ As a result of these and other position statements and articles, bone biopsy is suggested to categorize the following skeletal alterations: osteomalacia, adynamic bone disease, mixed uremic osteodystrophy, mild hyperparathyroidism, and osteitis fibrosa. In addition, no pathological aspects of bone are observed at times.^(^ ^24^ ^)^ However, the exact contribution of vitamin D deficiency to the underlying histological aspects of osteomalacia is difficult to ascertain and is rarely systematically assessed. For example, it has been reported that about 5% to 10% of patients on hemodialysis are diagnosed as having osteomalacia when bone biopsies are used in the evaluation.^(^ ^25^ ^)^ However, it should be noted that osteomalacia in the context of end‐stage renal disease can be caused by other factors such as aluminium intoxication^(^ ^26^ ^)^ or phosphate depletion.^(^ ^27^ ^)^

The prevalence of vitamin D deficiency in patients with chronic kidney and end‐stage renal disease is very high.^(^ ^28^ ^)^ Documented osteomalacia has occurred in patients with very low levels of 25OHD and central and peripheral fractures; furthermore, treatment with native vitamin D ameliorated histomorphometric indices evaluated by bone biopsy studies.^(^ ^29^ ^)^ These data highlight that vitamin D‐deficiency osteomalacia, in the context of the patient who is predialysis or on dialysis, is underdiagnosed and undertreated. Targeted studies are therefore needed to better identify the prevalence of osteomalacia in relation to other forms of skeletal alterations.

## Treatment

The goals of therapy for vitamin D‐deficiency osteomalacia are to alleviate symptoms, promote fracture healing, restore bone strength, and improve quality of life, while correcting biochemical abnormalities. There are no well‐established guidelines to guide therapy; most regimens are largely based on personal experience and the availability of suitable vitamin D preparations. Target levels of serum 25OHD should be aimed at maintaining >30 ng/mL^(^ ^1^, ^30^ ^)^ and PTH levels within the reference range. With effective therapy, clinical symptoms begin to improve within a few weeks; however, complete resolution of symptoms may take several months. Following treatment, it is a common finding to observe, at the beginning, an increase of serum alkaline phosphatase that gradually decreases; in patients with prolonged vitamin D deficiency, secondary hyperparathyroidism may persist for a long time, and in rare cases it may progress to hypercalcemic tertiary hyperparathyroidism. In that case, the increased PTH values are no more compensatory to the reduced serum calcium values but are a reflection of autonomous secretion of the hormone, irrespective of feedback mechanisms. Furthermore, depending on the amount of osteoid accumulation, a striking increase in BMD is observed, as seen after cure of osteomalacia in other conditions.^(^ ^31^ ^)^

Therapy consists of daily oral doses of vitamin D in the range of 800 to 1200 IU. Another schedule entails administration of 50 000 IU of native vitamin D weekly for 8 to 12 weeks, followed by a maintenance dose of 1000 to 2000 IU daily. Because these regimens may take a long time to reach vitamin D sufficiency, higher loading doses not exceeding 100 000 IU can be utilized based on pharmacological principles.^(^ ^32^ ^)^ Higher daily doses of vitamin D or alternatives routes^(^ ^33^ ^)^ may be necessary in cases of impaired GI absorption; in these circumstances up to 10,000 − 50,000 I. U. of native vitamin D can be utilized.

Treatment with vitamin D must be always accompanied by adequate calcium supplements. One‐thousand milligrams of elementary calcium divided in two or three doses is sufficient in most cases. Higher amounts in the range of 2000 to 3000 mg daily are needed in patients with malabsorption or after bariatric surgery, although poorly tolerated; this regimen can also reduce kidney stones in patients who have had gastric bypass surgery. In cases of malabsorption, calcifediol (wherever available) can be utilized because it is a more polar metabolite that is absorbed via the portal system. The use of calcitriol along with vitamin D may be preferred in cases of severe secondary hyperparathyroidism, in patients with concomitant calcium malabsorption or gastric bypass surgery, and in those with kidney failure. No studies are present in the literature directly addressing the cure of nutritional osteomalacia with head‐to‐head comparative studies of metabolites of vitamin D.

Vitamin D‐deficiency osteomalacia requires long‐term maintenance treatment that must be modulated according to the clinical and biochemical picture. Healing of osteomalacia and resolution of secondary hyperparathyroidism must be achieved.

For the specific conditions discussed above, it is clear that the treatment of osteomalacia cannot ignore the underlying primary disease. Use of another antiepileptic drug that does not interfere with vitamin D metabolism can be a choice. Treatment with vitamin D in the setting of renal failure should take into account the degree of suppression of PTH secretion, which may increase the risk of adynamic bone disease.

## Conclusion

There is a need for better understanding of the epidemiology of osteomalacia in both industrialized and developing countries. Information campaigns should be promoted to raise awareness of this neglected metabolic bone disease among specialists, nonspecialists, and general practitioners. It is important to recognize and appreciate, especially in the elderly, that a reduction in BMD cannot equate a diagnosis of osteoporosis. Bone biopsy studies of patients with specific diseases highly associated with the risk of developing osteomalacia should be carried out. The diseases are, for example, represented by conditions known to cause osteomalacia, not adequately responding to vitamin D treatment, some hypophosphatemic diseases and for a correct diagnosis of skeletal health in patients with kidney failure. Finally, owing to the inherent problems in performing bone biopsy procedures in clinical practice, a simplified tool should be developed that would help clinicians in the diagnosis of osteomalacia, similar to that proposed by the Japanese societies.^(^ ^4^ ^)^ However, this specific score should have international validation based on a concomitant bone‐histological diagnosis of osteomalacia.

## Disclosures

Prof Salvatore Minisola served as speaker for Abiogen, Amgen, Bruno Farmaceutici, Diasorin, Eli Lilly, Shire, Sandoz, and Takeda. He also served on the advisory boards of Abiogen, Kyowa Kirin, Pfizer, and UCB. All remaining authors declare no conflicts of interest.

## Author Contributions

**Salvatore Minisola:** Conceptualization; supervision; writing‐original draft; writing‐review and editing. **Luciano Colangelo:** Conceptualization; writing‐original draft; writing‐review and editing. **Jessica Pepe:** Conceptualization; writing‐original draft; writing‐review and editing. **DANIELE DIACINTI:** Conceptualization; writing‐original draft; writing‐review and editing. **Cristiana Cipriani:** Conceptualization; writing‐original draft; writing‐review and editing. **D. Sudhaker Rao:** Conceptualization; supervision; writing‐original draft; writing‐review and editing.

### Peer Review

The peer review history for this article is available at https://publons.com/publon/10.1002/jbm4.10447.
